# Evaluation of Sesame (*Sesamum indicum* L.) Genotypes for Agronomic Performance and Seed Quality Under Subtropical Australian Conditions

**DOI:** 10.3390/plants15182761

**Published:** 2026-09-09

**Authors:** Parbat Raj Thani, Andrew McDonald, Apurbo Kumar Chaki, Charissa Rixon, Mani Naiker, Tieneke Trotter

**Affiliations:** 1 School of Health, Medical and Applied Sciences, Central Queensland University, Rockhampton, QLD 4701, Australia; a.chaki@cqu.edu.au (A.K.C.); c.rixon@cqu.edu.au (C.R.); m.naiker@cqu.edu.au (M.N.); t.trotter@cqu.edu.au (T.T.); 2 AgriVentis Technologies Pty Ltd., Sydney, NSW 2000, Australia; amcdonald@agriventis.tech

**Keywords:** sesame, genotypes, Australian growing conditions, agronomic characteristics, yield attributes, total phenolic content, antioxidant activity, lignans, fatty acid

## Abstract

Sesame (*Sesamum indicum* L.) is an important oilseed crop valued for its oil, favourable fatty acid profile, and health-promoting phytoconstituents. However, information on the agronomic performance and seed-quality characteristics of sesame genotypes under Australian growing conditions remains limited. This study evaluated sesame genotypes under Australian conditions using complementary field evaluations at Kingaroy and Emerald, Queensland. At Kingaroy three genotypes (BLACK 2, WHITE 5, and WHITE 3) were evaluated across two growing seasons (2023/2024 and 2024/2025) to provide exploratory observations on oil yield and seed-quality characteristics. Because these genotypes were grown as single field strips and harvested seed was bulked within genotype, the Kingaroy data were treated as descriptive observations rather than statistically replicated genotype or seasonal effects. At Emerald, five genotypes (WHITE 6, BLACK 5, BLACK 2, WHITE 5, and WHITE 3) were evaluated using a randomised complete block design with four biological replicates, allowing statistical assessment of genotype effects on agronomic and seed-quality traits. Genotype significantly influenced crop establishment, first-pod height, screw-pressed oil yield, phytochemical composition, and fatty acid profile, whereas most other growth and yield traits did not differ significantly among genotypes. In the replicated Emerald experiment, final plant density ranged from 17.5 to 43.8 plants/m^2^, screw-pressed oil yield from 35.7 to 39.7%, total phenolic content (TPC), FRAP, and CUPRAC from 64.8 to 111.6 mg GAE/100 g DW, 87.3 to 124.8 mg TE/100 g DW, and 587.1 to 806.3 mg TE/100 g DW, respectively, and the sesamin and sesamolin from 107.9 to 281.5 mg/100 g DW and 96.6 to 138.9 mg/100 g DW, respectively. WHITE 3 generally showed high values for several phytochemical characteristics, including TPC, antioxidant capacity, and sesamin concentration. The Kingaroy evaluation showed numerical variation in oil yield and seed-quality characteristics across genotype–season combinations, providing complementary exploratory information but not statistical evidence of genotype or seasonal effects. Overall, the replicated Emerald experiment demonstrated substantial genotypic variation in several agronomic and seed-quality characteristics, highlighting the potential for genotype selection to improve sesame production and seed quality under Australian conditions. Further replicated multi-season and multi-location studies are required to assess the stability of these traits across Australian production environments.

## 1. Introduction

Sesame (*Sesamum indicum* L.) is one of the oldest cultivated oilseed crops and has long been recognised for its high nutritional and functional quality. Its seeds and oil are characterised by a high oil content, a favourable fatty acid profile, and a rich composition of bioactive constituents, particularly phenolic compounds and lignans [1]. Sesame oil is especially abundant in unsaturated fatty acids, mainly oleic and linoleic acids, which have been widely associated with beneficial effects on cardiovascular and metabolic health [2,3,4]. Sesame seeds also contain distinctive lignans, especially sesamin and sesamolin, which are recognised for their potential cholesterol-lowering effects, blood pressure regulation, and roles in lipid metabolism [5,6]. These compounds also possess antioxidant [2], and anti-inflammatory activities [7]. Together, these health-promoting properties highlight the importance of sesame as a functional food crop and a valuable raw material for the health-oriented food industry.

Beyond its nutritional value, sesame is an increasingly important commercial oilseed crop grown across tropical and subtropical environments [8,9,10]. The agronomic performance and phytochemical composition of sesame are strongly influenced by genotype, environmental conditions, and seasonal variation [9,11,12]. Traits such as plant growth, yield, yield components, oil content, fatty acid composition, and concentrations of health-promoting phytoconstituents can vary substantially across genotypes and production environments [13,14,15,16]. Such variation has direct implications for seed quality, productivity, and the commercial and nutritional value of sesame-based products [10,11,12]. Understanding genotypic variation in these traits under different production conditions is therefore important for identifying promising cultivars, informing breeding strategies, and supporting the development of sesame production across diverse growing regions [10,11,12,17].

Although numerous studies have evaluated sesame genotype performance in Asia, Africa, and South America, information on agronomic performance and seed quality under Australian growing conditions remains limited [1]. In Australia, the sesame market currently relies entirely on imports, while domestic interest in sesame production is increasing [18]. This situation highlights the need to evaluate sesame genotypes under Australian agro-climatic conditions to identify genotypes with strong agronomic performance and desirable quality attributes [1,18]. Such evaluations are particularly important because genotype responses observed under overseas production environments may not necessarily reflect performance under Australian conditions.

In this context, field evaluation of diverse sesame genotypes under Australian environments can provide information on both agronomic adaptation and seed-quality characteristics. Agronomic traits such as plant establishment, plant growth, yield components, and seed yield are important for assessing production potential, whereas oil yield, fatty acid composition, phenolic content, antioxidant capacity, and lignan concentration provide complementary information on the nutritional and functional quality of the harvested seed. Evaluating these characteristics together can therefore help identify genotypes that combine agronomic suitability with desirable seed-quality attributes.

The present study evaluated sesame genotypes under subtropical Australian conditions using complementary field experiments at Kingaroy and Emerald, Queensland. The Kingaroy evaluation involved three genotypes grown across two consecutive growing seasons and provided exploratory observations on oil yield, phytochemical composition, antioxidant capacity, lignan concentration, and fatty acid profile. Because the genotypes were grown as single field strips and the harvested seed was bulked within genotype, the Kingaroy evaluation was not designed to provide statistically replicated estimates of genotype or seasonal effects. In contrast, the Emerald experiment evaluated five sesame genotypes using a randomised complete block design with four biological replicates, providing the basis for statistical evaluation of genotype effects on crop establishment, growth, yield and yield components, oil yield, phytochemical composition, and fatty acid profile.

It was hypothesised that (i) sesame genotypes would differ in agronomic and seed-quality traits under Australian growing conditions; and (ii) genotypic differences would be evident in key seed-quality characteristics, including oil yield, phytochemical composition, lignan concentration, antioxidant capacity, and fatty acid composition. The Kingaroy evaluation was considered exploratory and provided descriptive observations across two growing seasons, whereas statistical inference regarding genotype effects was based on the replicated Emerald experiment. The study was therefore intended to identify genotypic variation in agronomic and seed-quality traits and provide a basis for subsequent replicated multi-season and multi-location evaluation of promising sesame genotypes under Australian production environments.

## 2. Results and Discussion

### 2.1. Effects of Genotype on Crop Establishment, Growth, Yield Components, and Yield

The five sesame genotypes, including the three genotypes (BLACK 2, WHITE 5, and WHITE 3) previously evaluated at the Kingaroy Research Facility, were grown at the Central Queensland Smart Cropping Centre during the 2025 growing season. The harvested seeds were subsequently analysed to evaluate the effects of genotype on crop establishment, growth, yield components, seed and oil yield, and phytochemical constituents.

#### 2.1.1. Effects on Crop Establishment

Crop establishment is a critical determinant of sesame productivity because it directly influences canopy development, resource capture, weed competitiveness, and ultimately yield formation [19]. After sowing seeds of each genotype (WHITE 6, BLACK 5, BLACK 2, WHITE 5, and WHITE 3) with equal seed rates (761,000 seeds/ha), the plant populations of these genotypes were measured at different stages, and the results are presented in Table 1. Across all genotypes, plant numbers gradually declined from 7 days after sowing (DAS) to harvest (99 DAS), reflecting the normal process of seedling mortality and plant attrition that occurs during crop establishment and subsequent crop development.

Significant differences in plant population were observed among the genotypes throughout the growing season. The genotype WHITE 6 consistently maintained the highest plant density, decreasing from 51.9 plants/m^2^ at 7 DAS to 43.8 plants/m^2^ at 99 DAS, while genotype WHITE 5 also exhibited strong establishment and stand persistence, declining from 47.5 to 38.1 plants/m^2^. In contrast, genotypes BLACK 2 and WHITE 3 recorded substantially lower populations throughout the assessment period, with final plant densities of only 17.5 and 22.5 plants/m^2^, respectively. These differences emerged as early as 7 DAS and remained relatively consistent until harvest, suggesting that genotype primarily influenced emergence and early establishment rather than survival during later developmental stages. Differences in emergence among genotypes are expected because in sesame, field emergence and seedling vigour are largely genetically controlled and heritable, and genotypes differ significantly in germination, emergence index, emergence rate index, and vigour traits, so genotypes can meaningfully influence stand establishment beyond laboratory germination alone [20,21,22].

An interesting observation from the present study is that genotypes with lower plant populations did not necessarily exhibit proportionally greater stand losses during crop development. Although the largest numerical reductions in plant population were observed in WHITE 5 (9.4 plants/m^2^) and WHITE 6 (8.1 plants/m^2^), these genotypes also possessed the highest initial populations. When stand persistence was expressed relative to the initial population, survival rates were remarkably similar among genotypes, ranging from 76.3% in BLACK 5 to 84.8% in BLACK 2. This indicates that differences in final plant density were primarily driven by establishment success rather than differential mortality after emergence. In other words, once seedlings became established, all genotypes exhibited relatively similar abilities to survive and complete their life cycle under the prevailing environmental conditions. Overall, these findings support previous reports that field emergence and final stand density in sesame are strongly influenced by genotype [21,22].

Overall, the data suggest that establishment is driven mainly by early vigour and emergence behaviour rather than by late-stage mortality. For Australian sesame production, that makes field establishment an important breeding and agronomic trait in its own right, because genotypes with more uniform emergence are likely to give more reliable crop stands, easier crop management, and yield stability across production environments.

#### 2.1.2. Effects on Growth Characteristics

The investigated sesame genotypes did not differ significantly in growth characteristics (plant height and number of side shoots) except for height to first pod (Table 2). Plant height ranged from 74.3 cm in genotype WHITE 3 to 87.1 cm in genotype WHITE 5, with an overall mean of 79.4 cm. The relatively small variation in plant height among genotypes suggests that vegetative growth was influenced more by the favourable growing environment than by genetic differences under the conditions of this study. Plant height is a quantitative trait regulated by multiple genes controlling cell division, internode elongation, and gibberellin-mediated stem growth [23,24], but its expression is also strongly modified by environmental factors such as temperature, soil moisture, nutrient availability, and solar radiation [25,26]. Consequently, the absence of significant genotypic differences suggests that the evaluated genotypes possessed comparable vegetative growth potential under the environmental conditions of the present study. Similar observations have been reported in sesame [24] and other oilseed crops [25], where environmental conditions frequently exert a greater influence on plant height than genetic factors, though significant genotype × environment interactions are commonly detected [25].

Although no significant differences in plant height were observed among the sesame genotypes evaluated in this study, considerable variation has been reported across different environments and genetic backgrounds worldwide. For example, Arpitha et al. [27] reported plant heights ranging from 80 to 146 cm among 270 sesame genotypes evaluated in India, while Bedawy and Mohamed [28] reported a mean plant height of 159.05 cm for 86 genotypes from Egypt, substantially higher than the values recorded in the present study. In contrast, Kıllı [29] reported plant heights ranging from 51.9 to 64.0 cm among 11 sesame genotypes in Turkey, which are lower than those observed in the current study.

The variation in plant height reported among different studies is expected because plant height development is highly responsive to environmental conditions and genetic backgrounds. Multiple studies have documented significant genotype-by-environment interactions in sesame, indicating that environmental factors substantially influence plant performance and morphological traits [30,31]. The observed phenotypic differences between genotypes reflect both inherent genetic potential and environmental influences in the tested locations [32]. Additionally, plant height has been identified as a key agronomic trait that varies significantly among sesame varieties, with both genetic and environmental factors contributing to this variation [24]. Therefore, direct comparison of plant height among experiments should be interpreted cautiously, as observed differences may reflect environmental influences in addition to genotypic variation.

The number of side shoots ranged from 1.0 to 1.9 per plant in genotypes WHITE 3 and WHITE 5, respectively, with an overall mean of 1.5 per plant, with no significant difference. Varying levels of shoot number have been reported in the literature. For example, Kıllı [29] reported shoot numbers per plant ranging from 1.5 to 3.5 in 11 different genotypes from Turkey, while Sumathi and Muralidharan [33] reported the number of shoots/plant in the range between 0.33 and 11.87 with an average value of 6.66 shoots/plant during the investigation of 30 hybrids and their 11 parent varieties in India. Branching is an important determinant of canopy architecture because side shoots increase the number of potential flowering sites and consequently the capacity for pod production [34]. However, greater branching does not necessarily result in higher seed yield, as excessive vegetative development may increase competition among branches for assimilates during reproductive growth [34,35]. Therefore, an optimum balance between vegetative growth and reproductive development is generally more beneficial than maximising branch number alone [36].

The height to the first pod varied from 26.0 cm in genotype BLACK 2 to 45.1 cm in genotype WHITE 5. Genotypes WHITE 6, BLACK 5, and WHITE 5 produced significantly higher first pod positions than genotype BLACK 2, while genotype WHITE 3 was intermediate. Among the growth characteristics evaluated, first pod height has particular agronomic importance because it directly influences the suitability of sesame for mechanised harvesting [37,38]. Plants with higher first pod insertion reduce the likelihood of pods remaining below the cutter bar during harvesting, thereby minimising harvest losses associated with low-positioned pods, as the height from soil to first pod must be higher than the blades of most combine harvesters to minimise pod loss [37]. The significantly greater first pod height observed in genotype WHITE 5 therefore represents a desirable architectural trait for commercial sesame production, particularly in mechanised farming systems where harvesting efficiency and reduced seed loss are major breeding objectives [38]. This diverse range of first pod heights has been reported in previous studies from across the world. For example, Eryigit et al. [39] have reported a first pod height in the range between 43.6 and 67.1 cm while investigating eight varieties of sesame in Turkey, and Ismaan et al. [40] have reported a range between 24.0 and 47.0 cm in six different varieties from Somalia.

Overall, from a breeding perspective, the limited variation observed in most vegetative growth characteristics suggests that further genetic improvement in sesame productivity may be achieved more effectively by targeting reproductive efficiency, seed quality, oil composition, and phytochemical accumulation rather than plant stature alone. Nevertheless, the superior first pod height of genotype WHITE 5 highlights its potential value as breeding material for improving harvestability in mechanised production systems. As commercial sesame production increasingly adopts mechanical harvesting, plant architectural traits such as first pod height are expected to become increasingly important selection criteria alongside yield and seed quality characteristics.

#### 2.1.3. Effects on Yield Attributes

The variation in yield attributes (pod number, seed density, and weight) in sesame genotypes was also reported to be nonsignificant (Table 2). The pod number varied from 37.9 pods/plant in genotype BLACK 5 to 55.7 pods/plant in genotype WHITE 6, with an average value of 45.6 pods/plant. The number of pods per plant is recognised as one of the principal yield components in sesame because it directly determines the potential number of seeds produced by each plant [41,42]. Pod production represents a cumulative outcome of branching capacity, flower initiation, pollination success, flower retention, pod set, and assimilate availability during reproductive development [43]. Consequently, variation in pod number reflects the combined influence of vegetative growth and reproductive efficiency rather than a single morphological characteristic. Although pod number did not differ significantly among the evaluated genotypes in the present study, the relatively narrow range observed suggests that reproductive development proceeded similarly under the favourable environmental conditions at Emerald. This may indicate that environmental conditions reduced the expression of genotypic differences in reproductive development, resulting in comparable pod formation across the evaluated germplasm.

Extensive genetic variation for pod number has nevertheless been reported in sesame germplasm. Sathish et al. [41] reported pod numbers ranging from 32 to 78 pods per plant with broad-sense heritability exceeding 70%, while Ismaila [44] also observed significant genotypic differences across multiple environments. Similarly, Golla [45] reported 27.89–44.17 pods/plant in three different varieties and Tesfaye et al. [46] reported 7.14–57.06 pods/plant in 300 different sesame varieties from Ethiopia. The higher value of pods/plant than the values observed in the present study has also been reported by several researchers, including Eryigit et al. [39], who have reported 56.1–125.5 pods/plant in eight varieties of sesame from Turkey. Compared with these reports, the relatively narrow range observed in the present study suggests that the evaluated Australian breeding lines exhibited comparable reproductive development under the prevailing environmental conditions.

Interestingly, despite the significant differences in plant establishment observed earlier, pod production per plant remained relatively similar among the evaluated genotypes. This pattern may indicate some degree of compensatory growth, whereby plants established at lower population densities could have benefited from reduced competition for light, water, nutrients, and growing space, potentially supporting reproductive development on a per-plant basis. Such compensatory responses have been reported in indeterminate crops such as sesame and may involve changes in branching, pod production per plant, or assimilate allocation to reproductive organs [47,48,49]. However, these mechanisms were not directly measured in the present study.

Seed bulk density ranged from 610.0 to 623.0 kg/m^3^, lower in genotypes BLACK 2 and WHITE 5 and higher in BLACK 5, with an average value of 614.1 kg/m^3^. Close values to the present study have been reported by several authors, including Garavand et al. [50] and Darvishi [51], who have reported seed bulk densities of 591.0 kg/m^3^ and 607.3 kg/m^3^, respectively in the sesame seeds with approximately 4.5% moisture content. Seed bulk density is an important physical quality parameter because it reflects the packing characteristics of seeds, which are influenced by seed size, shape, surface texture, and moisture content. In addition to its relevance for seed quality assessment, bulk density affects grain handling, transportation, storage efficiency, and processing performance [52,53].

Thousand-seed weight varied from 2.5 g/1000 seeds in genotype WHITE 3 to 2.8 g/1000 seeds in genotype WHITE 5, with an average value of 2.6 g/1000 seeds. Comparable weights to the present study have been reported in previous studies. For example, Tesfaye et al. [46] reported seed weight in the range between 1.66 and 3.33 g/1000 seeds in 300 different sesame varieties from Ethiopia. Eryigit et al. [39] have reported this value in the range between 2.85 and 3.67 g/1000 seeds in eight varieties of sesame from Turkey. Thousand-seed weight is an important yield component and can be influenced by resource availability during seed development [54]. The relatively narrow range of thousand-seed weight observed in this study suggests that seed size was relatively stable among the evaluated genotypes under the prevailing environmental conditions. Although seed weight may be associated with assimilate allocation during seed development, assimilate partitioning was not directly measured in the present study. Therefore, the observed similarity in thousand-seed weight should not be interpreted as direct evidence of similar physiological resource-allocation efficiency.

Overall, the limited variation observed in pod number, seed bulk density, and thousand-seed weight indicates that these yield attributes were comparatively stable among the evaluated genotypes, and suggests that any differences in final productivity were more likely to arise from cumulative physiological processes governing biomass production and assimilate partitioning than from variation in individual yield attributes.

#### 2.1.4. Effects on Yield (Biomass Production, Seed and Oil Yield, Harvest Index)

There were no significant differences in above-ground biomass, seed yield, and the harvest index among all the genotypes studied. The biomass production ranged from 3.4 tonne/ha DW in genotype WHITE 3 to 5.3 tonne/ha DW in genotype WHITE 6, with an overall mean of 4.3 tonne/ha DW. Above-ground biomass production represents the cumulative outcome of canopy development, light interception, radiation use efficiency, and dry matter accumulation throughout the growing season [55]. Consequently, biomass is often considered an integrative measure of crop growth because it reflects the overall efficiency with which plants convert intercepted solar radiation into vegetative tissues [55,56]. The absence of significant differences in biomass among the evaluated genotypes suggests that they possessed comparable capacities for canopy development and photosynthetic dry matter production under the prevailing environmental conditions at Emerald.

Siad et al. [57] have reported biomass yield in the range between 3.61 and 6.18 tonne/ha, while investigating six different varieties of sesame in Somalia. Sabikunnahar et al. [58] reported biomass in the range between 2.09 and 3.66 tonne/ha while investigating three varieties of sesame in Bangladesh. Variations in biomass reported among different studies are expected because biomass accumulation is strongly influenced by environmental conditions, including temperature, rainfall, solar radiation, soil fertility, and crop management practices [59,60]. Differences in growing season length and water availability may alter photosynthetic activity and canopy duration, ultimately affecting total dry matter production [59]. Therefore, comparisons among studies should consider both genotypic differences and environmental conditions under which crops were grown [60].

Clean hand-harvest seed yield ranged from 0.5 tonne/ha DW in genotype BLACK 5 to 0.9 tonne/ha DW in genotype WHITE 6, averaging 0.7 tonne/ha DW across genotypes. However, despite this numerical variation, no significant differences in seed yield were observed among genotypes. Seed yield is a complex quantitative trait resulting from the cumulative interaction of crop establishment, vegetative growth, flowering, pod retention, seed filling, and assimilate partitioning. The yield of sesame depends upon many independent contributing components, with pod number per plant, number of seeds per pod, and seed weight being the primary determinants [61]. Patel et al. [62] confirmed that pods per plant, seeds per pod, and biological yield per plant exhibited high heritability and substantial genetic gains. Consequently, variation in any single yield component does not necessarily result in proportional differences in final seed yield because plants often compensate through adjustments in other physiological processes. The absence of significant differences in seed yield among genotypes in the present study indicates that differences observed at earlier growth stages did not translate into significant differences in final seed yield under the conditions of the present study.

Remarkably, although significant differences in plant establishment were observed among the evaluated genotypes, these differences were not reflected proportionally in final seed yield. This pattern may be consistent with compensatory growth, whereby plants established at lower population densities could potentially benefit from greater availability of light, water, nutrients, and growing space [63]. Such responses may involve changes in branching, pod production per plant, or assimilate allocation to developing seeds. However, these physiological processes were not directly measured in the present study, and therefore the observed yield pattern should be interpreted as a possible indication of compensatory responses rather than direct evidence of the underlying mechanisms. Akter et al. [64] reported differences in dry matter partitioning and photosynthetic capacity among sesame genotypes, supporting the possibility that physiological compensation may contribute to yield stability under different plant populations.

Machine-harvest seed yield varied from 0.3 in genotype BLACK 2 to 0.5 tonne/ha DW in genotypes WHITE 5 and WHITE 3, with an overall mean of 0.4 tonne/ha DW. These values are slightly lower than the yield obtained from the hand-harvested seeds.

The harvest index ranged from 13.4% in genotype BLACK 5 to 18.2% in genotype WHITE 3. However, the differences in these values among genotypes were not statistically significant, suggesting broadly similar biomass allocation to seed production under the conditions of this experiment. The harvest index is an important indicator of crop productivity because it reflects the efficiency with which accumulated biomass is partitioned into the economic yield (seed) rather than vegetative tissues [65,66]. The absence of significant genotypic differences may be consistent with broadly similar biomass allocation to seed production among the evaluated genotypes under the prevailing environmental conditions [67]. However, assimilate partitioning was not directly measured in the present study. Diverse values of harvest index have been reported from across the world. Sabikunnahar et al. [58] have reported harvest index in the range between 22.4 and 23.2% while investigating three varieties of sesame, whereas Jahan and Abdullah-Al-Mamun [68] have reported these values in the range between 20.28% and 25.46% in six varieties of sesame from Bangladesh. Similarly, Ismaan et al. [40] reported a much wider range of 13.93–40.94% across six sesame varieties grown in Somalia. The differences between the present study and previous reports are likely attributable to variations in genotype, environmental conditions, and crop management practices [65,69]. Harvest index is strongly influenced by both genetic and environmental factors [65], with water availability being particularly critical for sesame, as demonstrated by the significant effects of irrigation regimes on harvest index performance [70].

Screw-pressed oil yield differed numerically among genotypes, ranging from 34.0% in BLACK 5 to 39.7% in BLACK 2. Genotype BLACK 2 produced the highest oil recovery, followed by WHITE 3 (37.6%) and WHITE 5 (36.7%), while BLACK 5 had the lowest oil extraction efficiency. These results indicate that genotypes with moderate seed yield may still possess superior oil production potential due to higher oil concentration or extractability. The variation in oil yield among genotypes highlights the importance of considering both seed productivity and oil recovery when selecting sesame cultivars for commercial production.

Overall, except for first pod height and screw-pressed oil yield, genotype had little effect on growth, yield, and yield-related traits under the environmental conditions of this study. The similarity in biomass production, seed yield, and harvest index indicates broadly comparable agronomic performance among the evaluated genotypes under the specific Emerald environment. Although significant differences in crop establishment resulted in considerable variation in plant population among genotypes, these differences were not reflected in proportional changes in plant growth, biomass accumulation, or seed yield. For example, despite establishing the highest plant population, WHITE 6 did not produce a significantly greater seed yield than WHITE 5, which had a comparatively lower plant density. This pattern may be consistent with phenotypic plasticity or compensatory responses, whereby plants growing at lower population densities could have benefited from reduced competition for available resources. Such responses may involve adjustment in plant growth, reproductive development, or assimilate allocation; however, these underlying mechanisms were not directly measured in the present study. Further physiological measurements would be required to determine the mechanisms responsible for the observed stability in seed yield.

### 2.2. Effects of Genotype on Phytochemical Composition

Although most of the agronomic characteristics were not significantly different, substantial genotypic variation was observed for the measured phytochemical characteristics under the Emerald experiment (Table 3). Total phenolic content (TPC) ranged from 64.8 mg gallic acid equivalents (GAE)/100 g dry weight (DW) in genotype BLACK 5 to 111.6 mg GAE/100 g DW in genotype WHITE 3, with an overall mean of 91.2 mg GAE/100 g DW. The TPC values observed in the Emerald experiment were within the range reported for sesame genotypes in many previous studies. For example, Hoyos et al. [1] reported TPC in the range between 89.3 and 146.7 mg GAE/100 g while analysing 11 different sesame varieties in Australia. However, others have reported higher TPC values than the values observed in the present study. For example, Kurt et al. [71] collected 17 different sesame genotypes from nine different geographical regions across the world and grew them in Turkey, investigated TPC in them and reported values ranging between 199.0 mg GAE/100 g and 681.0 mg GAE/100 g. Furthermore, Osei et al. [72] evaluated 112 different genotypes from Africa and Asia and reported TPC in the range between 498.0 and 8788 mg/100 g. Similarly, Dossou et al. [73] reported TPC variation from 272 to 2198 mg GAE/100 g across 400 sesame varieties.

The antioxidant capacity measured by ferric reducing antioxidant capacity (FRAP) and cupric reducing antioxidant capacity (CUPRAC) assays in the present study ranged from 87.3 mg Trolox equivalents (TE)/100 g DW to 124.8 mg TE/100 g DW and 587.1 to 806.3 mg TE/100 g DW, with an average value of 112.2 mg TE/100 g DW and 747.8 mg TE/100 g DW, respectively. The FRAP and CUPRAC values observed in the present study were comparable with the previous report by Hoyos et al. [1], who have reported FRAP values in the range between 53.5 mg TE/100 g DW and 195.2 mg TE/100 g DW and the CUPRAC values in the range between 465.99 mg TE/100 g DW and 2969.56 mg TE/100 g DW for the 11 different genotypes grown in Australia.

Such wide variations in published TPC, FRAP, and CUPRAC values are unlikely to be attributable to genotype alone. Differences among studies may also reflect seed colour, maturity stage, climatic conditions, extraction solvent and extraction time, as well as the analytical approach used for the Folin–Ciocalteu, FRAP, and CUPRAC assays and whether results are expressed on a dry-weight or fresh-weight basis [74,75,76,77,78,79]. Therefore, comparisons among published values should be interpreted cautiously because biological variation and methodological differences can both contribute to the observed range.

Furthermore, the values of both antioxidant measurement methods followed a similar trend to TPC, with the genotype WHITE 3 showing the highest FRAP and CUPRAC, while BLACK 5 recorded the lowest FRAP and CUPRAC values. The relatively close agreement between TPC, FRAP and CUPRAC rankings indicates that samples with higher TPC also tended to show higher antioxidant-capacity values. However, because the present study did not isolate individual phenolic compounds or experimentally determine their contribution to antioxidant activity, this association should not be interpreted as direct evidence that phenolics were the principal biochemical determinants of antioxidant capacity.

Sesamin concentration varied from 107.9 to 281.5 mg/100 g seed DW, with an overall mean of 174.2 mg/100 g seed DW. The highest sesamin content was observed in WHITE 3, whereas BLACK 5 contained the lowest amount. Sesamolin concentration ranged from 96.6 mg/100 g seed DW to 138.9 mg/100 g seed DW, with an overall mean of 116.9 mg/100 g seed DW. The genotype WHITE 3 produced the highest sesamolin concentration, while WHITE 6 recorded the lowest concentration. Diverse values of sesamin and sesamolin have been reported among sesame genotypes worldwide, with some values comparable to those observed in the present study and others considerably higher [1,80,81,82]. For example, Hoyos et al. [1] reported sesamin ranging from 36.0 mg/100 g to 256.0 mg/100 g and sesamolin ranging from 46.0 mg/100 g to 167.0 mg/100 g in 11 different genotypes in Australia, with both ranges encompassing the values observed in the present study. On the other hand, Wang et al. [80] examined sesamin and sesamolin concentrations in 62 different sesame genotypes in China and observed values ranging from 82.0 mg/100 g to 1105 mg/100 g, and 135.0 to 696.0 mg/100 g, respectively. The broad range reported across studies indicates substantial variation in lignan accumulation among sesame genotypes.

The three sesame genotypes (BLACK 2, WHITE 5, and WHITE 3) were also grown at the Kingaroy Research Facility, Queensland, across two growing seasons (2023/2024 and 2024/2025) and the harvested seeds were assessed for oil yield, TPC, antioxidant capacity (FRAP and CUPRAC), and lignans (sesamin and sesamolin). However, because seeds were bulked within genotype and independent field replicates were not available, these measurements are presented as descriptive observations in Table A1. Across the evaluated Kingaroy genotype–season combinations, variation was observed in oil yield (37.1 to 40.0%), TPC (74.7–88.3 mg GAE/100 g DW), FRAP (91.9–129.4 mg TE/100 g DW), CUPRAC (540.1–673.3 mg TE/100 g DW), sesamin (184.7–212.1 mg/100 g DW), and sesamolin (81.4–116.3 mg/100 g DW) contents. These values should be interpreted as comparative observations under the tested conditions rather than as statistically supported estimates of genotype effects.

Among the Kingaroy observations, BLACK 2 exhibited numerically higher TPC, FRAP, and CUPRAC values (88.3 mg GAE/100 g DW, 129.4 mg TE/100 g DW, and 673.3 mg TE/100 g DW, respectively), whereas WHITE 3 showed comparatively lower values (74.7 mg GAE/100 g DW, 91.9 mg TE/100 g DW, and 540.1 mg TE/100 g DW, respectively). These observations were consistent with the higher antioxidant capacity observed in samples with higher TPC. Similar positive relationships between phenolic concentration and antioxidant activity have been reported in sesame and other oilseed crops [83]. Furthermore, although BLACK 2 showed higher TPC and antioxidant capacity, WHITE 5 contained the highest concentrations of sesamin (212.1 mg/100 g DW) and sesamolin (116.3 mg/100 g DW). This disparity indicates that lignan concentrations did not follow the same numerical pattern as TPC and antioxidant capacity in the evaluated samples. The finding is consistent with our previous study, which demonstrated that sesamin and sesamolin possess relatively weak reducing power and make a limited contribution to TPC and antioxidant capacity, even when present at elevated concentrations [84].

Across the two growing seasons, screw-pressed oil yield showed little numerical variation (38.1–38.4%), whereas the phytochemical measurements showed greater seasonal differences. The mean TPC, FRAP, CUPRAC, sesamin, and sesamolin concentrations ranged from 78.8 to 87.8 mg GAE/100 g DW, 102.6–126.3 mg TE/100 g DW, 598.1–616.8 mg TE/100 g DW, 193.9–204.7 mg/100 g DW, and 97.7–99.9 mg/100 g DW, respectively. Across all measured phytochemical parameters, the 2024/2025 growing season generally showed higher numerical values than the 2023/2024 season. Because the Kingaroy experiment did not include independent biological field replication within season, these differences are presented as descriptive seasonal observations rather than statistically supported seasonal effects.

Seasonal differences may reflect differences in environmental conditions during seed development and maturation. Temperature, solar radiation, water availability, and other abiotic factors can influence secondary metabolism and the accumulation of phenolic and antioxidant compounds [74,75,85]. These processes provide a plausible explanation for the higher numerical phytochemical values observed in 2024/2025; however, the present study did not isolate the effects of individual environmental variables or measure the underlying metabolic responses. Therefore, this interpretation should be considered a possible explanation rather than a demonstrated mechanism.

The magnitude and direction of seasonal differences also varied among genotypes for some phytochemical traits. Similar variation in phytochemical concentrations among sesame genotypes and growing years has been reported previously [86,87]. However, because the Kingaroy experiment lacked independent biological field replication, these observations do not provide a statistically supported test of genotype × season interaction. Rather, they indicate that genotype-associated differences in phytochemical composition may vary across growing seasons, a possibility that should be evaluated in replicated multi-season and multi-location experiments.

Overall, the Emerald experiment provides the statistical basis for evaluating genotype effects on phytochemical composition under Australian production conditions. The significant genotypic variation observed for TPC, antioxidant capacity, and lignan concentration demonstrates that the evaluated sesame genotypes differed in their capacity to accumulate bioactive metabolites under the same environmental conditions. This finding highlights the importance of genetic factors in regulating secondary metabolism and suggests that substantial improvements in seed nutritional quality may be achieved through genotype selection. Interestingly, these marked differences in phytochemical composition occurred despite relatively small differences in several agronomic traits among genotypes, suggesting that phytochemical characteristics may provide useful additional criteria for genotype selection. The Kingaroy evaluation provides useful descriptive observations across two growing seasons, including numerical variation in TPC, antioxidant capacity, and lignan concentrations. However, these observations should not be interpreted as evidence of stable genotype performance, seasonal effects, or genotype × environment interactions. The independently replicated Emerald experiment provides the appropriate basis for statistical inference regarding genotype effects. The Kingaroy observations therefore complement the replicated Emerald findings by identifying patterns that warrant further evaluation in replicated multi-season and multi-location experiments.

The fatty acid composition of sesame seeds from different genotypes grown at Emerald is presented in Table 4. Four major fatty acids were identified: two saturated fatty acids (SFAs), palmitic acid (C16:0) and stearic acid (C18:0); one monounsaturated fatty acid (MUFA), oleic acid (C18:1 *n*-9); and one polyunsaturated fatty acid (PUFA), linoleic acid (C18:2 *n*-6). Several minor fatty acids, including eicosanoic, behenic, and lignoceric acids, were also detected; however, their combined contribution was below 0.5%, and therefore they were not quantified in this study. Significant genotypic variation in fatty acid composition was observed among the five sesame genotypes. Palmitic acid ranged from 9.1 to 10.9%, stearic acid from 5.7 to 6.4%, total SFA from 15.6 to 16.9%, oleic acid from 38.4 to 40.7%, linoleic acid from 43.1 to 45.6%, total UFA from 83.0 to 84.3%, and the UFA/SFA ratio from 4.9 to 5.4. Among the genotypes, BLACK 2 again exhibited the lowest SFA concentrations and the highest UFA, and UFA/SFA ratio, and the genotype WHITE 5 recorded the highest SFA concentrations and the lowest UFA and UFA/SFA ratio.

Differences in seed fatty-acid composition among sesame genotypes have been reported previously and are consistent with genetic variation in fatty-acid biosynthesis and desaturation pathways. Seed oil is stored mainly as triacylglycerols, and the final proportions of palmitic, stearic, oleic, and linoleic acids depend on enzymes in the fatty-acid synthesis and desaturation pathways, including stearoyl-ACP desaturase, FAD2, FAD3, FatB, and DGAT. Natural variation in these pathways is associated with differences in seed-oil profiles, and different genotypes can therefore maintain similar total oil content while still differing in fatty-acid composition because oil quantity and fatty-acid profile are only partly coupled by shared genetic and regulatory control [88,89,90]. These mechanisms provide a plausible biological basis for genotype-associated variation in fatty-acid composition; however, the present Kingaroy experiment did not include independent biological field replication and therefore cannot establish that the numerical differences observed among the genotype-level samples were caused by genotype.

The fatty acid composition observed in the present study agrees closely with previous reports for sesame grown under diverse agroecological conditions [91,92,93,94,95,96]. For instance, Matthäus and Özcan [92] reported palmitic and stearic acid concentrations ranging from 6.06 to 9.03% and 5.29–6.42%, respectively, while Kurt [91] reported corresponding ranges of 8.19–10.26% and 4.63–6.35%. Similarly, oleic acid concentrations observed in the present study (38.5–40.0%) fall well within the range of 36.13–43.63% reported by Kurt [91] for Turkish and international sesame varieties. Linoleic acid concentrations (43.5–45.2%) were likewise consistent with the values (39.13–46.38%) reported across 24 sesame cultivars by the same author [91]. Across the evaluated genotype-level samples, total UFAs accounted for 83.1–84.2% of the total fatty acid composition, confirming that sesame oil is predominantly composed of unsaturated fatty acids. Comparable levels of unsaturated fatty acids have been reported previously by Kurt [91], Zangui et al. [94] and other researchers [95,96], indicating that the fatty-acid profile observed in the present study was broadly consistent with previously reported sesame oil profiles. The UFA/SFA ratio varied from 4.9 to 5.3 among the evaluated genotype-level samples, which is comparable with previously reported values [91]. This ratio is widely regarded as an important indicator of nutritional quality because higher values reflect a greater proportion of unsaturated fatty acids relative to saturated fatty acids. Diets rich in unsaturated fatty acids are associated with many health-benefiting activities, such as cardiovascular health [97,98]. Sesame oil is therefore considered nutritionally attractive because it combines high oleic and linoleic acid levels with relatively low saturated-fat content.

The fatty acid composition of the sesame genotypes from the Kingaroy experiment in two different years, 2023/2024 and 2024/2025, was also evaluated and the results are presented in Table A2. As shown, genotypic differences were observed for palmitic acid (9.2–10.9%), stearic acid (5.9–6.6%), total saturated fatty acids (SFA; 15.8–16.9%), oleic acid (38.5–40.0%), linoleic acid (43.5–45.2%), total unsaturated fatty acids (MUFA + PUFA or UFA; 83.1–84.2%), and the UFA/SFA ratio (4.9–5.3). The genotype BLACK 2 showed the most unsaturated profile, with the lowest SFA, and the highest UFA, and UFA/SFA ratio, while the genotype WHITE 5 was the opposite extreme, with the highest SFA, and the lowest UFA/SFA ratio. Because the Kingaroy samples were derived from single field strips and lacked independent biological field replication, these differences are presented as descriptive observations rather than statistically supported genotype effects.

The numerical differences in fatty acid composition (palmitic acid, stearic acid, total SFA, oleic acid, linoleic acid, and total unsaturated fatty acids) were comparatively small between 2023/2024 and 2024/2025. Consequently, the UFA/SFA ratio declined only marginally from 5.1 to 5.0, indicating that the relative balance between unsaturated and saturated fatty acids was similar between the two growing seasons. However, because the Kingaroy experiment did not include independent biological field replication within each season, these observations cannot be used to determine whether seasonal differences were statistically significant or to quantify the relative contributions of genotype and season.

Fatty acid biosynthesis occurs primarily during the seed-filling stage, during which environmental factors such as temperature, solar radiation, and water availability can affect the activity of key desaturase enzymes responsible for converting saturated fatty acids into unsaturated fatty acids. However, the environmental variation between the two growing seasons in the present study was associated with relatively small numerical differences in the fatty-acid profiles. Previous studies have shown that environmental conditions during seed development can alter fatty-acid composition, particularly through effects on fatty-acid desaturation [88,89,90,99,100]. The relatively similar profiles observed between the two Kingaroy seasons may therefore reflect the environmental conditions experienced during seed development, but the present experiment did not measure desaturase activity, gene expression, or other physiological responses that would allow this mechanism to be evaluated directly.

The genotype-level samples also showed some variation in the magnitude and direction of seasonal changes for individual fatty acids. For example, oleic acid increased from 38.8 to 39.3% in BLACK 2 and from 39.7 to 40.3% in WHITE 3 between the two seasons, whereas it remained essentially unchanged in WHITE 5. Such patterns are consistent with the possibility that fatty-acid composition may respond differently among genotypes under contrasting seasonal conditions. However, because each genotype was represented by a single field strip at Kingaroy, these observations cannot be interpreted as a statistically demonstrated genotype × season interaction. Replicated multi-season and multi-location experiments are required to determine whether such genotype-specific seasonal responses are consistent.

Overall, the replicated Emerald experiment demonstrated significant genotypic variation in the fatty acid composition of sesame oil, particularly for SFA, UFA, and the UFA/SFA ratio. The Kingaroy evaluation provided complementary descriptive observations across two growing seasons and showed that the overall fatty acid profile remained relatively stable numerically between seasons. However, the Kingaroy observations cannot be used to establish genotype effects, seasonal effects, or genotype × season interactions because independent biological field replication was not available. Further replicated multi-season and multi-location experiments would be valuable for determining the stability of fatty acid profiles across environments.

### 2.3. Correlation

The potential correlations among different variables were investigated using Pearson’s R correlation analysis, and the results are presented in Table 5. The study revealed significant relationships among agronomic characteristics, antioxidant properties, lignans and fatty acid composition of sesame genotypes.

Among the agronomic traits, plant height was positively correlated with the number of side shoots (*r* = 0.592, *p* < 0.01), indicating that taller plants generally produced more branches. Biomass production also exhibited a strong positive correlation with side shoots (*r* = 0.791, *p* < 0.01) and a moderate positive correlation with the number of pods per plant (*r* = 0.493, *p* < 0.05), indicating that genotypes or experimental units with greater branching also tended to have greater biomass and pod number. Seed yield was strongly associated with biomass (*r* = 0.757, *p* < 0.01), indicating that higher biomass was associated with higher seed yield in the present dataset. The harvest index showed a positive relationship with seed yield (*r* = 0.512, *p* < 0.05) but was negatively correlated with side shoots (*r* = −0.450, *p* < 0.05) and bulk density (*r* = −0.545, *p* < 0.05), indicating that greater side-shoot number and bulk density were associated with lower harvest index in this dataset. These results are consistent with previous sesame breeding studies [101,102]. For example, Dinkar et al. [101] reported positive relationships among branches, pods, biological yield, harvest index, and seed yield in 22 sesame varieties. Similarly, Mohanty et al. [102] also found positive associations between seed yield and plant height, biological yield, and oil yield per plant.

TPC exhibited a strong positive association with FRAP (*r* = 0.640, *p* < 0.01), while FRAP was also strongly correlated with CUPRAC (*r* = 0.880, *p* < 0.01). Furthermore, TPC showed a significant positive relationship with CUPRAC (*r* = 0.532, *p* < 0.05). These strong associations indicate that samples with higher TPC also tended to exhibit greater antioxidant capacity in both FRAP and CUPRAC assays. Many previous studies have similarly reported positive associations between TPC and antioxidant activity in sesame [72,103,104,105].

Sesamin demonstrated significant positive correlations with screw-pressed oil (*r* = 0.587, *p* < 0.01) and FRAP (*r* = 0.545, *p* < 0.05), and similarly, sesamolin was positively associated with screw-pressed oil (*r* = 0.459, *p* < 0.05), FRAP (*r* = 0.646, *p* < 0.01) and sesamin (*r* = 0.737, *p* < 0.01). Similar results have been reported by many other authors [1,81,106,107]. For example, Moazzami and Kamal-Eldin [106] and Hoyos et al. [1] have reported a positive correlation between sesamin and sesamolin. A positive correlation between oil content and lignan content was also reported by Kancharla and Arumugam [81]. This association is consistent with the present finding that higher screw-pressed oil yield tended to coincide with higher lignan concentrations. A weak positive correlation between lignans and TPC or antioxidant activity was reported by Kouighat et al. [108], which suggests that the two major lignans accumulate simultaneously and contribute, at least partially, to antioxidant activity.

Significant correlations were also observed among fatty acids. Palmitic acid (C16:0) showed a strong positive correlation with total saturated fatty acids (SFA) (*r* = 0.948, *p* < 0.01) and a strong negative correlation with stearic acid (C18:0) (*r* = −0.800, *p* < 0.01). Since palmitic acid constitutes the major saturated fatty acid in sesame, variations in SFA are primarily determined by changes in palmitic acid concentration. Similar compositional relationships have been reported by Dar et al. [107].

Oleic acid (C18:1 *n*-9) exhibited a significant negative correlation with linoleic acid (C18:2 *n*-6) (*r* = −0.870, *p* < 0.01). Similar results have been reported by several researchers, including Dar et al. [107] and Were et al. [109]. This inverse relationship may result from enzymatic conversion and seed-specific regulation of FAD2, where microsomal oleate desaturase converts oleic acid to linoleic acid [110,111].

Unsaturated fatty acids (UFAs) were strongly and negatively correlated with SFA (*r* = −0.978, *p* < 0.01), while exhibiting positive relationships with the UFA/SFA ratio (*r* = 0.975, *p* < 0.01). Similarly, the UFA/SFA ratio showed an almost perfect negative association with SFA (*r* = −0.988, *p* < 0.01). These relationships are mathematically expected because UFA and SFA together comprise the total fatty acid pool, and the UFA/SFA ratio is directly determined by their relative proportions.

Overall, the correlation analysis indicates that seed yield was primarily driven by biomass production rather than individual plant morphological traits. Antioxidant capacity was more closely associated with total phenolic compounds than with lignan concentrations, supporting previous observations that sesamin and sesamolin contribute only modestly to the total antioxidant potential of sesame. The strong inverse relationship between oleic and linoleic acids further confirms the coordinated regulation of fatty acid desaturation pathways in sesame seeds, while the negative association between saturated and unsaturated fatty acids reflects the expected compositional balance of seed oil. These findings provide valuable insights for breeding programmes aimed at simultaneously improving seed yield, antioxidant properties and oil quality.

## 3. Materials and Methods

### 3.1. Agronomy

#### 3.1.1. Trial Location, Crop Establishment, and Soil

Five genotypes (WHITE 6, WHITE 3, WHITE 5, BLACK 5, and BLACK 2) of sesame were acquired from AgriVentis Technologies Pty Ltd. ((https://www.agriventistechnologies.com.au) (1 Macquarie Place, Sydney, NSW, Australia)), details of which are described in Table 6, were selected for the present study. Field experiments were conducted at two locations in Queensland, Australia.

The first experiment was established at the Kingaroy Research Facility in southeast Queensland (26.58° S, 151.83° E). Three genotypes (BLACK 2, WHITE 5, and WHITE 3) were grown during two consecutive growing seasons, with sowing on 14 December 2023 and harvest on 2 May 2024 for the 2023/2024 season, and sowing on 3 December 2024 and harvest on 23 May 2025 for the 2024/2025 season. Each genotype was grown in a linear strip, consisting of two rows (230 m in length) spaced 50 cm apart, under uniform field management in both growing seasons. The soil at the site was classified as a Ferrosol, and sowing was carried out using a PPS planter at a seeding rate of 700,000 seeds/ha in both growing seasons. The harvested seed from each genotype was bulked to obtain a representative genotype-level sample for subsequent laboratory analyses of oil yield, fatty acid composition, TPC, antioxidant capacity, and lignan concentration. Laboratory analyses were performed using technical replicates to ensure analytical precision and reproducibility. Because independent replicated field plots were not available, the Kingaroy data are presented as comparative observations of genotype and seasonal performance under the tested conditions rather than as estimates of within-site biological variability.

A second field experiment was conducted at Emerald (23.52° S, 148.20° E) during the 2025 growing season to evaluate the effects of five sesame genotypes (WHITE 6, WHITE 3, WHITE 5, BLACK 5, and BLACK 2) on growth, agronomic performance, seed and oil yield, and the composition of health-promoting phytoconstituents. Seeds for these genotypes were obtained from the Kingaroy Research Facility, Katherine Research Station (Northern Territory), and the Central Queensland Smart Cropping Centre (Queensland). The soil was classified as a Vertosol, and the experiment was arranged in a randomised complete block design (RCBD) with four biological replicates. The experimental area was divided into four beds representing two field positions (upper and lower sections of the field) to account for potential spatial variability across the site. Each genotype was randomly allocated once within each replicate.

Plots were established using a precision planter equipped with a 120-08 seed plate and five knockers. Seeds were sown on 21 October 2025 at a target depth of approximately 2.5 cm, or into available soil moisture where appropriate. A target plant population of approximately 761,000 seeds/ha was used. The planter was operated at a ground speed of 6–8 km/h with cog settings of 14 teeth on Cog A and 24 teeth on Cog B.

#### 3.1.2. Crop and Weather Conditions

The climatic data for the sesame growing season in Emerald are presented in Table 7, and the climatic data for Kingaroy for both years, presented in Table A3, were obtained from the Australian Government Bureau of Meteorology (http://www.bom.gov.au, accessed on 10 May 2026).

#### 3.1.3. Weed and Nutrient Management

Basal fertiliser was applied at sowing using Superfect Potash 3:1 at 500 kg/ha. Nitrogen was supplied as urea at a rate of 180 kg/ha. Standard agronomic practices for weed, pest and disease management were implemented throughout the growing season to ensure optimum crop establishment and growth. Plants reached physiological maturity on 28 January 2026 and were harvested at 99 days after sowing.

#### 3.1.4. Data Collection

During crop emergence, a 1 m section of the middle row from each plot was marked and was the designated data collection point for each plot. Plant population, plant height, biomass yield, and hand-harvested seed yield were all collected from this data collection point.

##### Plant Population

The number of emerged plants was counted and recorded randomly from a 1 m section of row in each plot at 7, 17, 31, and 99 DAS. The plant population (plants/m^2^) was then calculated using a given formula as described (Equation (1)).

(1) $$Plant\;{Population\;(Plants/m}^{2})=\frac{Number\;of\;plants\;in\;1\;m\;of\;row}{Row\;space\;(0.4\;m)}$$

##### Plant Height, Number of Pods per Plant, Biomass Yield, and Hand-Harvested Seed Yield

All plants from the data collection point within each plot were hand-harvested at 99 days by cutting plants at ground level with secateurs. Five of the harvested plants were selected at random, and the total height of each of the five plants was measured and recorded, and the number of pods on each of the five plants was counted and recorded [112]. The total plant material cut was then weighed to calculate total biomass (Equation (2)) before being threshed with a Kimseed CW09 Thresher (Wangara, WA, Australia) to extract the seed. The seed was cleaned with a Kimseed MK3 Seed Cleaner (Wangara, WA, Australia) and the cleaned seed weight was recorded. A 5 g subsample was then taken and dried at 76 °C until stable, and seed moisture was calculated on a dry weight basis (Equation (3)). The seed moisture and the cleaned seed weight were then used to calculate the hand-harvested seed yield in tonne/ha at 6% moisture (Equation (4)).

(2) $$Biomass\left(tonne/ha\right)=\frac{Total\;plant\;material\;cut\;\left(kg\right)}{Row\;lentght\;*\;number\;of\;rows\;*\;0.4}\;*\;10$$

where 0.4 m represents the fixed row spacing of the plot, and 10 is the conversion factor for converting yield from kg/m^2^ to tonnes per hectare (tonne/ha).

(3) $$Moisture\;\%=\frac{Fresh\;Weight-Dry\;Weight}{Dry\;Weight}$$

(4) $$Seed\;Yield\;(tonne/ha)=(\frac{Sample\;Weight\left(g\right)}{Row\;length\;\left(m\right)\;*\;number\;of\;rows\;*\;0.4})\;*\;0.01\;*\;\left(\frac{1-\%\;Seed\;Moisture}{Dry\;weight\;(0.94\%)}\right)$$

where 0.4 m represents the fixed row spacing of the plot, 0.01 is the conversion factor for converting yield from g/m^2^ to tonne/ha, and 0.94 represents the assumed dry matter fraction corresponding to a standard seed moisture content of 6%.

##### Machine-Harvested Seed Yield

After the hand-harvest was completed, a 10 m length of bed (5 rows) was harvested with a plot header (Zurn 150, Zurn Harvesting GmbH & Co. KG, Schöntal-Westernhausen, Germany). The machine-dressed seed was then cleaned and the weight recorded and the seed moisture determined using the equipment and methods described in Section [Plant Height, Number of Pods per Plant, Biomass Yield, and Hand-Harvested Seed Yield]. The machine-harvested yield at 6% moisture was then calculated (Equation (4)).

##### Seed Bulk Density and 1000 Seed Weight

The cleaned machine-harvested seed was used to determine the seed bulk density and the 1000 seed weight. The bulk density was determined by filling a Graintec Scientific 500 mL Chondrometer canister and recording the weight of the seed in grams; the density was reported as kg/m^3^.

A small sample of the cleaned machine-harvested seed was placed in a seed counter, and 1000 seeds were counted three times to ensure accuracy. After the third counting, the seed was weighed and the weight in grams per 1000 seeds was recorded [112].

##### Harvest Index (HI)

Harvest index (HI) was calculated as the ratio of manually harvested seed yield to total above-ground biomass and expressed as a percentage [113] (Equation (5)).

(5) $$HI\;\%=\frac{Dry\;seed\;Yield}{Dry\;abovegroung\;biomass}\times 100$$

### 3.2. Phytochemistry

#### 3.2.1. Chemicals and Reagents

All analytical-grade chemicals and reagents used in this study were obtained from ChemSupply (Gillman, SA, Australia) or Sigma-Aldrich (Melbourne, VIC, Australia). Milli-Q^®^ water was used for the preparation of all solutions and throughout the chemical analyses. Prepared reagents and chemical solutions were stored at 4 °C in the dark until use.

#### 3.2.2. Extraction Protocol

The sesame seed samples were extracted using a methanolic extraction protocol developed in our laboratory and previously adopted by multiple studies [1,83,114,115]. Freeze-dried seeds were milled to a fine powder using a Breville grinder (BCG200) for 1 min. Approximately 0.5 g of the powdered material was combined with 7 mL of 90% (*v*/*v*) methanol in a centrifuge tube, vortex-mixed for 10 s, and agitated on an end-over-end shaker (Ratek RM4) at 50 rpm for 60 min. The extract was centrifuged at 1000× *g* for 10 min using a Heraeus X1 Multifuge (Thermo Fisher Scientific, Melbourne, VIC, Australia), and the supernatant was recovered. The remaining pellet was subjected to a second extraction with a fresh 7 mL aliquot of 90% methanol, agitated for 20 min, and centrifuged under identical conditions. The resulting supernatants were pooled, brought to a final volume of 14 mL with 90% methanol, and stored at 4 °C in the dark until further analysis.

#### 3.2.3. Experimental Analysis

##### Screw-Pressed Oil Yield

Sesame seeds were screw-pressed, and oil yield for the different genotypes was determined following a protocol developed in our laboratory [84,114]. In brief, 20 g of sesame seeds were processed using an automatic oil press (Wgwioo; 110/220 V, 600–1500 W, 42 × 16 × 32 cm^3^) operated at 150 °C, with a constant shaft speed of 58 rpm and a feeding rate of 20 g/min. Seed moisture content was carefully controlled, as it is known to significantly influence oil recovery [116,117,118,119,120]. Based on previous findings indicating optimal oil quality at a moisture content of 5.3% and a worm-shaft speed of 45 rpm, seed moisture was adjusted to approximately 5.3% using a mass balance calculation (Equation (6)). The seeds were then screw-pressed, and the extracted oil was collected in 10 mL centrifuge tubes and centrifuged at 3000× *g* for 20 min to separate the clarified oil from the sediment. The clear oil layer was carefully transferred into fresh centrifuge tubes, while residual oil remaining in the original tubes was recovered by rinsing with *n*-hexane, leaving the sediment behind. Oil yield was then calculated according to Equation (7).

(6) $$Mass\;ballance\;equation=\frac{\left(target\;moisture\;\%-initial\;moisture\;\%\right)}{100}\times seed\;weight$$

(7) $$Oil\;yield\;(\%)=\frac{\left(weight\;of\;oil\right)}{\left(seed\;weight\;used\;for\;screw\;press\;extraction\right)}\times 100$$

##### TPC

TPC was determined using the Folin–Ciocalteu method as described in our previous study [83]. Briefly, 400 µL of diluted extract was mixed with 2 mL of diluted Folin–Ciocalteu reagent (1:10, *v*/*v*) and incubated in the dark at room temperature for 10 min. After adding 2 mL of 7.5% (*w*/*v*) sodium carbonate, the mixture was incubated at 40 °C for 30 min, and absorbance was measured at 760 nm using a UV–Vis spectrophotometer (Thermo Scientific Genesys 10S UV–Vis Spectrophotometer, Madison, WI, USA) with Milli-Q^®^ water as the blank. TPC was quantified using a gallic acid standard curve (20–100 mg/L) and expressed as mg GAE/100 g DW.

##### Antioxidant Capacity

The FRAP assay measures the ferric reducing capacity of the extract and provides an estimate of its electron-donating antioxidant activity. This assay was performed according to the method described in our previous study [83]. Briefly, the FRAP working reagent was freshly prepared by mixing acetate buffer, TPTZ (2,4,6-tris(2-pyridyl)-s-triazine) solution, and ferric chloride solution at a ratio of 10:1:1 (*v*/*v*/*v*). An aliquot of 100 µL of sample extract was mixed with 3 mL of the FRAP reagent and incubated at 37 °C for 4 min. The absorbance was then measured at 593 nm using a UV–Vis spectrophotometer (Thermo Scientific Genesys 10S UV–Vis Spectrophotometer, Madison, WI, USA). Antioxidant capacity was expressed as mg Trolox equivalents (TE)/100 g DW based on a Trolox calibration.

The CUPRAC assay complements FRAP by evaluating the cupric ion reducing capacity of antioxidants under near-neutral pH conditions. This assay was performed according to the method described in our previous study [83]. Briefly, the CUPRAC reagent consisted of 10 mM CuCl_2_, 1 M ammonium acetate buffer, and 7.5 mM neocuproine prepared in ethanol. Equal volumes (1 mL each) of the CuCl_2_ solution, ammonium acetate buffer, and neocuproine solution were mixed with 1 mL of Milli-Q^®^ water and 100 µL of sample extract. The reaction mixture was incubated at 50 °C for 30 min, after which the absorbance was measured at 450 nm using a UV–Vis spectrophotometer (Thermo Scientific Genesys 10S UV–Vis Spectrophotometer, Madison, WI, USA). Antioxidant capacity was calculated from a Trolox calibration curve (50–500 mg/L) and expressed as mg Trolox equivalents (TE)/100 g DW.

##### Lignans

The lignans (sesamin and sesamolin) in seed extracts were quantified by high-performance liquid chromatography, using an Agilent 1100 system (G1313A autosampler, G1322A vacuum degasser, G1311A quaternary pump, and G1365B multi-wavelength detector module; Agilent Technologies, Santa Clara, CA, USA), following the procedures applied by Hoyos et al. [1]. Briefly, separation was achieved on an Eclipse XDB-C18 column with an isocratic water:methanol mobile phase (20:80, *v*/*v*) at 0.8 mL/min. Samples (10 µL) were analysed for 10 min at room temperature with ultraviolet detection at 287 nm. Lignans were identified by comparison with authentic standards and quantified using external calibration curves (0–300 ppm), with results expressed as mg/100 g DW. The calibration equations, linearity (R^2^), limits of detection (LOD), and limits of quantification (LOQ) for sesamin were Y = 18.828x − 85.8, 0.9969, 19.2 mg/L, and 58.3 mg/L, respectively, while the corresponding values for sesamolin were Y = 15.874x + 86.68, 0.9988, 12.0 mg/L, and 36.4 mg/L, respectively.

##### Fatty Acids

Fatty acid methyl esters (FAMEs) were prepared following the procedures applied in our previous studies [83]. Briefly, 0.02 g of oil was saponified with 0.4 M NaOH in methanol at 55 °C for 1.5 h, followed by hexane extraction. The hexane layer was washed, filtered, diluted, and stored at 4 °C until analysis.

FAMEs were analysed by gas chromatography–mass spectrometry (Shimadzu QP2010 Plus, Kyoto, Japan) equipped with a Restek FAMEWAX column (Restek Corporation, Bellefonte, PA, USA) (30 m × 0.32 mm I.D. × 0.25 µm thickness). Samples (0.5 µL) were injected in split mode (split ratio = 10) at 250 °C with helium as the carrier gas at a flow rate of 2 mL/min. The oven temperature was programmed from 195 to 240 °C with a total run time of 35 min for each sample. FAMEs were identified using mass spectra and authenticated standards, and the fatty acids were quantified as a percentage of the total identified fatty acids in the oil.

### 3.3. Statistical Analysis

For the Emerald experiment, the individual field plot was considered the experimental unit, with four independent biological replicates per genotype (*n* = 4), and the sample of each biological replicate was analysed in the laboratory using technical replicates. The technical replicate measurements were not treated as independent experimental units and were averaged within each biological replicate before statistical analysis. For the Kingaroy experiment, representative bulked seed samples obtained from each genotype were analysed using technical replicates to ensure analytical precision. Oil yield was determined using three technical replicates per biological replicate, whereas all other biochemical variables were analysed using two technical replicates per biological replicate. Results are presented as mean ± standard error (SE).

Statistical analyses were performed for Emerald samples using IBM SPSS Statistics Version 28.0. A linear mixed-effects model was used, with genotype included as a fixed effect and block included as a random effect to account for variation associated with the RCBD. Prior to analysis, the assumptions of normality and homogeneity of variance were assessed using the Shapiro–Wilk and Levene’s tests, respectively. When a significant genotype effect was detected (*p* < 0.05), genotype means were compared using Sidak-adjusted pairwise comparisons. Pearson’s correlation analysis was performed using the Emerald dataset to evaluate the relationships among the measured variables.

## 4. Conclusions

This study demonstrated substantial variation among sesame genotypes in agronomic performance, seed quality, phytochemical composition, antioxidant capacity, lignan concentration, and fatty acid profile under Australian growing conditions. At Kingaroy, descriptive differences among genotypes were observed for oil yield, TPC, antioxidant capacity, lignan concentration, and fatty acid composition. Phytochemical values were generally higher in the 2024/2025 season than in 2023/2024, while fatty acid composition was comparatively stable. However, because each genotype was represented by a single field strip per season and harvested seed was bulked within genotype, these Kingaroy observations should be considered preliminary and exploratory rather than statistically supported genotype, seasonal, or genotype × seasonal effects.

The Emerald trial revealed considerable variation in crop establishment among genotypes; however, these differences were not translated into significant differences in biomass production, seed yield, or most agronomic traits. Nevertheless, the absence of significant differences in seed yield and biomass indicates broadly comparable agronomic performance among the evaluated genotypes under the specific Emerald conditions.

Despite the limited variation in yield performance, substantial differences in phytochemical composition were observed. The replicated Emerald experiment provided evidence that genotype was associated with variation in several seed-quality and phytochemical traits, including oil yield, TPC, antioxidant capacity, and lignan concentration. These genotype-related differences were more pronounced for seed-quality traits than for seed yield.

Overall, the findings highlight the importance of considering both agronomic and quality traits when selecting sesame cultivars for Australian production, particularly where value-added oil and phytochemical traits are important selection criteria.

Although seed yield differences among the evaluated genotypes were relatively small, variation in oil yield, antioxidant capacity, lignan concentration, and fatty acid composition indicates opportunities for targeted selection according to specific end-use quality attributes. However, claims regarding suitability for premium oils, functional foods, or nutraceutical applications require further evaluation of product performance, stability, and nutritional or functional properties.

Further replicated multi-location and multi-season trials are required to determine the stability of genotype performance across Australian environments and to formally evaluate genotype × environment interactions. Such studies should include independent biological field replication at each environment and season and should incorporate additional environments representative of the major sesame-growing regions of Australia.

## Figures and Tables

**Table 1:** Plant population (plants/m^2^) of five sesame genotypes at different crop growth stages at Emerald, Queensland, Australia.

Genotype	Plant Population (plants/m^2^) on Different Days After Sowing			
	7 Days	17 Days	31 Days	99 Days
WHITE 6	51.9 ± 2.8 ^c^	49.4 ± 5.7 ^b^	46.9 ± 6.7 ^b^	43.8 ± 5.1 ^c^
BLACK 5	36.9 ± 6.2 ^ab^	33.8 ± 5.2 ^ab^	32.5 ± 4.7 ^ab^	28.1 ± 3.3 ^ab^
BLACK 2	20.6 ± 1.6 ^a^	18.8 ± 3.0 ^a^	18.8 ± 3.3 ^a^	17.5 ± 2.7 ^a^
WHITE 5	47.5 ± 2.7 ^b^	46.3 ± 3.3 ^b^	44.4 ± 2.6 ^b^	38.1 ± 1.9 ^bc^
WHITE 3	26.9 ± 3.6 ^a^	25.0 ± 3.2 ^a^	22.5 ± 4.4 ^a^	22.5 ± 3.7 ^a^
Average	36.8 ± 3.1	34.6 ± 3.2	33.0 ± 3.2	30.0 ± 2.6
*p*-value	<0.001	<0.001	0.001	<0.001

The results are presented as mean ± SE based on four biological replicate measurements (*n* = 4) per genotype. Superscript letters indicate Sidak-adjusted pairwise comparisons of genotype-estimated marginal means. Genotypes sharing at least one superscript letter are not significantly different, whereas genotypes with no common letter differ significantly (*p* < 0.05). A three-colour scale (red, white, and green) in the table indicates the lowest (17.5 plants/m^2^), intermediate (34.7 plants/m^2^), and the highest (51.9 plants/m^2^) values, respectively 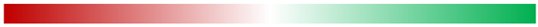.

**Table 2:** Growth, yield and yield-contributing components of five sesame genotypes grown at Emerald, Queensland, Australia.

Genotype	Plant Height (cm)	Height to First Pod (cm)	Side Shoots	Pods/Plant	Seed Bulk Density (kg/m^3^)	Seed Weight (g/1000 seeds)	Biomass (tonne/ha DW)	Clean Hand-Harvest Seed Yield (tonne/ha DW)	Clean Machine-Harvest Seed Yield (tonne/ha DW)	Harvest Index (%)	Screw-Pressed Oil Yield % DW
WHITE 6	80.8 ± 4.5 ^a^	43.8 ± 1.0 ^b^	1.8 ± 0.5 ^a^	55.7 ± 15.9 ^a^	614.0 ± 6.1 ^a^	2.6 ± 0.1 ^a^	5.3 ± 0.9 ^a^	0.9 ± 0.1 ^a^	0.4 ± 0.1 ^a^	17.9 ± 0.8 ^a^	35.3 ± 1.2 ^a^
BLACK 5	75.9 ± 4.0 ^a^	40.8 ± 4.0 ^b^	1.3 ± 0.5 ^a^	37.9 ± 4.7 ^a^	623.0 ± 5.3 ^a^	2.6 ± 0.1 ^a^	3.6 ± 0.3 ^a^	0.5 ± 0.1 ^a^	0.4 ± 0.1 ^a^	13.4 ± 2.7 ^a^	35.7 ± 0.6 ^a^
BLACK 2	78.9 ± 4.7 ^a^	26.0 ± 3.3 ^a^	1.8 ± 0.5 ^a^	48.1 ± 8.1 ^a^	610.0 ± 2.4 ^a^	2.6 ± 0.1 ^a^	4.4 ± 0.8 ^a^	0.6 ± 0.0 ^a^	0.3 ± 0.0 ^a^	15.3 ± 2.8 ^a^	39.7 ± 0.9 ^b^
WHITE 5	87.1 ± 3.6 ^a^	45.1 ± 1.3 ^b^	1.9 ± 0.4 ^a^	38.1 ± 7.6 ^a^	610.0 ± 1.6 ^a^	2.8 ± 0.0 ^a^	4.7 ± 0.6 ^a^	0.8 ± 0.1 ^a^	0.5 ± 0.0 ^a^	16.7 ± 1.8 ^a^	38.0 ± 0.7 ^ab^
WHITE 3	74.3 ± 5.8 ^a^	32.6 ± 4.9 ^ab^	1.0 ± 0.2 ^a^	48.1 ± 8.9 ^a^	613.5 ± 5.1 ^a^	2.5 ± 0.1 ^a^	3.4 ± 0.3 ^a^	0.6 ± 0.1 ^a^	0.5 ± 0.1 ^a^	18.2 ± 3.0 ^a^	38.1 ± 0.8 ^ab^
Average	79.4 ± 2.1	37.6 ± 2.1	1.5 ± 0.2	45.6 ± 4.2	614.1 ± 2.1	2.6 ± 0.0	4.3 ± 0.3	0.7 ± 0.1	0.4 ± 0.0	16.3 ± 1.0	37.4 ± 0.4
*p*-value	0.353	0.004	0.535	0.665	0.276	0.379	0.242	0.096	0.278	0.606	0.04

Results are presented as mean values ± SE based on four independent biological replicates (*n* = 4) per genotype. For screw-pressed oil yield, each biological replicate was analysed in triplicate using three laboratory technical replicates, which were averaged for each biological replicate before statistical analysis. Superscript letters indicate significant differences among genotype-estimated marginal means based on Sidak-adjusted pairwise comparisons. Genotypes sharing at least one superscript letter are not significantly different, whereas genotypes with no common letter differ significantly (*p* < 0.05).

**Table 3:** TPC, antioxidant capacity, sesamin, and sesamolin concentrations of five sesame genotypes grown at Emerald, Queensland, Australia. TPC, total phenolic content; FRAP, ferric reducing antioxidant power; CUPRAC, cupric reducing antioxidant capacity; GAEs, gallic acid equivalents; TEs, Trolox equivalents; DW, dry weight.

Genotype	TPC (mg GAE/100 g DW)	FRAP (mg TE/100 g DW)	CUPRAC (mg TE/100 g DW)	Sesamin (mg/100 g DW)	Sesamolin (mg/100 g DW)
WHITE 6	104.7 ± 1.2 ^cd^	109.1 ± 2.0 ^b^	782.0 ± 8.6 ^b^	119.1 ± 3.1 ^a^	96.6 ± 1.9 ^a^
BLACK 5	64.8 ± 4.8 ^a^	87.3 ± 1.8 ^a^	587.1 ± 23.2 ^a^	107.9 ± 6.6 ^a^	107.4 ± 2.8 ^b^
BLACK 2	79.5 ± 1.4 ^b^	115.3 ± 2.7 ^b^	765.6 ± 36.0 ^b^	229.1 ± 8.9 ^b^	116.0 ± 1.9 ^c^
WHITE 5	95.7 ± 1.1 ^c^	124.4 ± 1.9 ^c^	797.9 ± 32.6 ^b^	133.3 ± 3.7 ^a^	125.7 ± 1.0 ^d^
WHITE 3	111.6 ± 1.9 ^d^	124.8 ± 2.3 ^c^	806.3 ± 29.3 ^b^	281.5 ± 9.5 ^c^	138.9 ± 1.8 ^e^
Average	91.2 ± 2.9	112.2 ± 2.4	747.8 ± 17.6	174.2 ± 11.4	116.9 ± 2.5
*p*-value	<0.001	<0.001	<0.001	<0.001	<0.001

Results are presented as mean values ± SE based on four independent biological replicates (*n* = 4) per genotype. Each biological replicate was analysed in duplicate using two laboratory technical replicates, which were averaged for each biological replicate before statistical analysis. Superscript letters indicate Sidak-adjusted pairwise comparisons of genotype-estimated marginal means. Genotypes sharing at least one superscript letter are not significantly different, whereas genotypes with no common letter differ significantly (*p* < 0.05).

**Table 4:** Fatty acid composition (% of total identified fatty acids) of screw-pressed oil from five sesame genotypes grown at Emerald, Queensland, Australia. SFAs, saturated fatty acids; MUFAs, monounsaturated fatty acids; PUFAs, polyunsaturated fatty acids; UFAs, unsaturated fatty acids; UFA/SFA, ratio of total unsaturated to saturated fatty acids.

Genotype	Palmitic Acid (C16:0)	Stearic Acid (C18:0)	Total SFA	Oleic Acid (C18:1 *n*-9)	Linoleic Acid (C18:2 *n*-6)	UFA	UFA/SFA
WHITE 6	10.4 ± 0.1 ^c^	6.0 ± 0.0 ^b^	16.5 ± 0.1 ^c^	39.7 ± 0.1 ^b^	43.6 ± 0.2 ^b^	83.4 ± 0.1 ^b^	5.0 ± 0.0 ^ab^
BLACK 5	9.6 ± 0.0 ^b^	6.4 ± 0.1 ^c^	16.0 ± 0.1 ^b^	40.7 ± 0.0 ^d^	43.1 ± 0.1 ^a^	83.9 ± 0.1 ^c^	5.2 ± 0.0 ^c^
BLACK 2	9.1 ± 0.0 ^a^	6.4 ± 0.0 ^c^	15.6 ± 0.0 ^a^	38.7 ± 0.0 ^a^	45.6 ± 0.0 ^d^	84.3 ± 0.0 ^d^	5.4 ± 0.0 ^d^
WHITE 5	10.9 ± 0.1 ^d^	5.9 ± 0.0 ^ab^	16.9 ± 0.1 ^d^	38.4 ± 0.1 ^a^	44.5 ± 0.0 ^c^	83.0 ± 0.1 ^a^	4.9 ± 0.0 ^a^
WHITE 3	10.6 ± 0.1 ^c^	5.7 ± 0.0 ^a^	16.3 ± 0.1 ^bc^	40.2 ± 0.1 ^c^	43.3 ± 0.1 ^ab^	83.6 ± 0.1 ^bc^	5.1 ± 0.0 ^bc^
Average	10.1 ± 0.2	6.1 ± 0.1	16.3 ± 0.1	39.6 ± 0.2	44.0 ± 0.2	83.6 ± 0.1	5.1 ± 0.0
*p*-value	<0.001	<0.001	<0.001	<0.001	<0.001	<0.001	<0.001

Results are presented as mean values ± SE based on four independent biological replicates (*n* = 4) per genotype. Superscript letters indicate Sidak-adjusted pairwise comparisons of genotype-estimated marginal means. Genotypes sharing at least one superscript letter are not significantly different, whereas genotypes with no common letter differ significantly (*p* < 0.05).

**Table 5:** Pearson correlation coefficients among agronomic, seed quality, phytochemical, and fatty acid variables measured in the Emerald experiment.

	Plant ht	First Pod ht	Side Shoots	Pods/ Plant	Bulk Density	Seed Weight	Biomass	Seed Yield	Harvest Index	Screw Pressed Oil	TPC	FRAP	CUPRAC	Sesamin	Sesamolin	C16:0	C18:0	SFA	C18:1 *n*-9	C18:2 *n*-6	UFA
First pod ht	0.398																				
Side shoots	0.592 **	−0.002																			
Pods/plant	0.192	−0.375	0.509 *																		
Bulk density	0.325	0.339	0.305	−0.058																	
Seed weight	0.240	0.179	0.325	0.283	0.085																
Biomass	0.326	0.011	0.791 **	0.493 *	0.064	0.209															
Seed yield	0.162	0.096	0.364	0.409	−0.294	0.145	0.757 **														
Harvest index	−0.185	0.004	−0.450 *	−0.023	−0.545 *	−0.134	−0.144	0.512 *													
Screw pressed oil	0.158	−0.388	0.036	−0.129	−0.217	−0.260	−0.124	−0.163	0.034												
TPC	0.036	0.123	−0.152	0.173	−0.357	−0.047	0.062	0.415	0.461 *	0.043											
FRAP	0.097	−0.140	−0.001	−0.016	−0.555 *	0.155	−0.019	0.227	0.366	0.391	0.640 **										
CUPRAC	0.017	−0.065	0.000	−0.044	−0.511 *	0.067	0.013	0.258	0.384	0.195	0.532 *	0.880 **									
Sesamin	−0.136	−0.603 **	−0.233	0.095	−0.210	−0.264	−0.330	−0.233	0.128	0.587 **	0.400	0.545 *	0.362								
Sesamolin	−0.089	−0.244	−0.319	−0.203	−0.263	0.032	−0.462 *	−0.268	0.139	0.459 *	0.394	0.646 **	0.359	0.737 **							
C16:0	0.153	0.536 *	−0.031	−0.079	−0.161	0.244	0.084	0.344	0.248	−0.182	0.701 **	0.500 *	0.430	−0.104	0.310						
C18:0	−0.110	−0.189	0.129	−0.066	0.150	−0.189	0.093	−0.194	−0.301	0.028	−0.752 **	−0.646 **	−0.521 *	−0.346	−0.533 *	−0.800 **					
SFA	0.167	0.670 **	−0.030	−0.212	−0.138	0.217	0.082	0.312	0.198	−0.251	0.548 *	0.343	0.310	−0.328	0.175	0.948 **	−0.582 **				
C18:1 *n*-9	−0.405	0.088	−0.339	−0.053	0.446 *	−0.298	−0.313	−0.302	−0.101	−0.488 *	−0.149	−0.579 **	−0.463 *	−0.063	−0.123	−0.111	0.040	−0.116			
C18:2 *n*-6	0.292	−0.406	0.393	0.163	−0.342	0.183	0.333	0.177	−0.036	0.603 **	−0.158	0.359	0.270	0.184	0.006	−0.347	0.281	−0.359	−0.870 **		
UFA	−0.207	−0.690 **	0.088	0.235	0.127	−0.220	0.002	−0.263	−0.228	0.291	−0.562 **	−0.350	−0.308	0.306	−0.204	−0.932 **	0.621 **	−0.978 **	0.128	0.370	
UFA/SFA	−0.204	−0.676 **	0.018	0.175	0.098	−0.183	−0.063	−0.289	−0.180	0.279	−0.557 *	−0.311	−0.293	0.329	−0.138	−0.937 **	0.580 **	−0.988 **	0.091	0.386	0.975 **

** Correlation is significant at the 0.01 level; * correlation is significant at the 0.05 level; the sample size of each variable was *n* = 20 independent field experimental units (five genotypes × four biological replicates); a three-colour scale (red, white, and green) was used to represent correlations of −1, 0, and 1, respectively. 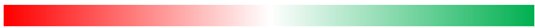.

**Table 6:** Description of Australian sesame genotypes used in this study.

Sr. No.	Genotype	Description
1	WHITE 6	White seed, long season, non-shattering sesame. Up to 170 days to harvest. Developed in Israel.
2	BLACK 5	Black seed, long season, capsules in pairs, non-shattering sesame. Up to 170 days to harvest. Preferred for shiny black seed. Developed in Texas, USA.
3	BLACK 2	Black seed, medium season, capsules in triplets, non-shattering sesame. Standard 135 days to harvest. Developed in Israel.
4	WHITE 5	White seed, medium-long season, capsules in pairs, non-shattering sesame. Developed in Texas, USA.
5	WHITE 3	White seed, a quick variety with a short season, capsules in triplets, non-shattering sesame. Developed in Texas, USA.

**Table 7:** Average monthly climatic conditions during the sesame growing season at Emerald, Queensland, Australia.

Months	Precipitation Monthly Total (mm)	Lowest Daily Solar Exposure (MJ m^−2^)	Highest Daily Solar Exposure (MJ m^−2^)	Monthly Mean Solar Exposure (MJ m^−2)^	Lowest Daily Temp (°C)	Highest Daily Temp (°C)	Monthly Mean Temperature (°C)
October 2025	22.4	16.7	27.8	25.0	31.8	40.4	35.8
November 2025	43.2	6.1	30.3	24.9	26.3	42.0	36.0
December 2025	8.4	12.0	30.8	25.8	30.7	39.1	35.7
January 2026	192.8	7.2	30.6	22.9	26.5	37.3	33.6

## Data Availability

The original contributions presented in this study are included in the article. Further inquiries can be directed to the corresponding author .

## Appendix A

**Table A1:** Descriptive comparison of screw-pressed oil yield and phytochemical characteristics of three sesame genotypes evaluated at Kingaroy, Queensland, across two growing seasons (2023/2024 and 2024/2025). TPC, total phenolic content; FRAP, ferric reducing antioxidant power; CUPRAC, cupric reducing antioxidant capacity; GAEs, gallic acid equivalents; TEs, Trolox equivalents; DW, dry weight.

Genotype	Oil Yield (% DW of Seed)			TPC (mg GAE/100 g DW)			FRAP (mg TE/100 g DW)			CUPRAC (mg TE/100 g DW)			Sesamin (mg/100 g DW)			Sesamolin (mg/100 g DW)		
	2023/2024	2024/2025	Mean	2023/2024	2024/2025	Mean	2023/2024	2024/2025	Mean	2023/2024	2024/2025	Mean	2023/2024	2024/2025	Mean	2023/2024	2024/2025	Mean
BLACK 2	40.6 ± 2.3	39.4 ± 2.0	40.0 ± 1.4	86.3 ± 0.7	90.2 ± 0.7	88.3 ± 1.2	120.1 ± 0.2	138.6 ± 5.5	129.4 ± 5.8	686.4 ± 8.6	660.2 ± 3.9	673.3 ± 8.5	174.4 ± 0.7	195.0 ± 0.4	184.7 ± 6.0	92.3 ± 0.4	105.4 ± 0.3	98.8 ± 3.8
WHITE 5	37.8 ± 2.1	36.4 ± 1.8	37.1 ± 1.3	79.6 ± 1.4	94.3 ± 4.2	87.0 ± 4.6	110.7 ± 1.8	133.6 ± 1.2	122.2 ± 6.7	602.6 ± 4.6	615.1 ± 11.4	608.9 ± 6.2	198.6 ± 0.9	225.6 ± 0.1	212.1 ± 7.8	117.5 ± 0.8	115.0 ± 0.3	116.3 ± 0.8
WHITE 3	36.9 ± 2.1	38.4 ± 2.2	37.7 ± 1.4	70.5 ± 0.5	79.0 ± 0.2	74.7 ± 2.4	77.1 ± 1.9	106.7 ± 1.9	91.9 ± 8.6	505.2 ± 3.7	575.1 ± 6.3	540.1 ± 20.4	208.8 ± 1.8	193.3 ± 1.3	201.1 ± 4.6	83.5 ± 0.3	79.4 ± 0.7	81.4 ± 1.2
Average	38.4 ± 1.2	38.1 ± 1.1		78.8 ± 2.9	87.8 ± 3.1		102.6 ± 8.3	126.3 ± 6.5		598.1 ± 33.2	616.8 ± 15.9		193.9 ± 6.5	204.7 ± 6.6		97.7 ± 6.5	99.9 ± 6.7	

Results for each year are presented as average values ± SE (*n* = 3 technical replicates for oil yield, while *n* = 2 technical replicates for other variables).

**Table A2:** Descriptive comparison of fatty acid composition (% of total identified fatty acids) of screw-pressed oil from three sesame genotypes evaluated at Kingaroy, Queensland, across two growing seasons (2023/2024 and 2024/2025). SFAs, saturated fatty acids; MUFAs, monounsaturated fatty acids; PUFAs, polyunsaturated fatty acids; UFAs, unsaturated fatty acids; UFA/SFA, ratio of total unsaturated to saturated fatty acids.

Genotype	Palmitic Acid (C16:0)			Stearic Acid (C18:0)			Total SFA			Oleic Acid (C18:1 *n*-9)			Linoleic Acid (C18:2 *n-*6)			UFA			UFA/SFA		
	2023/2024	2024/2025	Mean	2023/2024	2024/2025	Mean	2023/2024	2024/2025	Mean	2023/2024	2024/2025	Mean	2023/2024	2024/2025	Mean	2023/2024	2024/2025	Mean	2023/2024	2024/2025	Mean
BLACK 2	9.2 ± 0.0	9.2 ± 0.0	9.2 ± 0.0	6.4 ± 0.0	6.8 ± 0.0	6.6 ± 0.1	15.6 ± 0.0	16.1 ± 0.0	15.8 ± 0.1	38.8 ± 0.0	39.3 ± 0.0	39.0 ± 0.1	45.6 ± 0.0	44.7 ± 0.0	45.2 ± 0.3	84.4 ± 0.0	83.9 ± 0.0	84.2 ± 0.1	5.4 ± 0.0	5.2 ± 0.0	5.3 ± 0.0
WHITE 5	10.9 ± 0.1	10.8 ± 0.1	10.9 ± 0.1	6.0 ± 0.0	6.2 ± 0.1	6.1 ± 0.1	16.9 ± 0.2	17.0 ± 0.2	16.9 ± 0.1	38.5 ± 0.1	38.5 ± 0.0	38.5 ± 0.0	44.6 ± 0.1	44.6 ± 0.2	44.6 ± 0.1	83.1 ± 0.2	83.0 ± 0.2	83.1 ± 0.1	4.9 ± 0.1	4.9 ± 0.1	4.9 ± 0.0
WHITE 3	10.6 ± 0.1	10.5 ± 0.1	10.5 ± 0.1	5.8 ± 0.0	6.1 ± 0.0	5.9 ± 0.1	16.4 ± 0.1	16.6 ± 0.2	16.5 ± 0.1	40.3 ± 0.1	39.7 ± 0.1	40.0 ± 0.2	43.3 ± 0.2	43.7 ± 0.3	43.5 ± 0.2	83.6 ± 0.1	83.4 ± 0.2	83.5 ± 0.1	5.1 ± 0.0	5.0 ± 0.1	5.1 ± 0.0
Average	10.2 ± 0.3	10.2 ± 0.3		6.1 ± 0.1	6.4 ± 0.2		16.3 ± 0.2	16.5 ± 0.2		39.2 ± 0.4	39.1 ± 0.2		44.5 ± 0.4	44.3 ± 0.2		83.7 ± 0.2	83.5 ± 0.2		5.1 ± 0.1	5.0 ± 0.1	

Results for each year are presented as average values ± SE (*n* = 2 technical replicates).

**Table A3:** Average monthly climatic conditions during the sesame growing season in Kingaroy, Queensland, Australia.

Months	Precipitation Monthly Total (mm)		Lowest Daily Solar Exposure (MJ m^−2^)		Highest Daily Solar Exposure (MJ m^−2^)		Monthly Mean Solar Exposure (MJ m^−2)^		Lowest Daily Temp (°C)		Highest Daily Temp (°C)		Monthly Mean Temperature (°C)	
	2023/24	2024/25	2023/24	2024/25	2023/24	2024/25	2023/24	2024/25	2023/24	2024/25	2023/24	2024/25	2023/24	2024/25
December	115.4	271.8	11.1	9.6	31.3	31.4	26.9	24.1	26.3	23.1	36.1	35.5	32.0	29.8
January	140.6	131.8	7.5	12.2	30.2	31.0	22.1	24.8	25.1	25.2	37.3	34.4	30.8	29.6
February	50.6	40.8	7.4	11.5	28.7	28.1	22.4	23.2	23.4	25.1	33.8	31.4	30.0	29.2
March	128.4	97.4	5.9	6.3	26.3	26.2	17.6	16.9	19.7	20.9	32.8	32.7	27.0	27.9
April	96.0	54.8	5.0	3.7	20.4	20.8	16.5	16.2	17.8	20.6	29.9	27.5	25.0	25.2
May	15.0	68.4	9.4	6.4	16.6	17.5	13.8	13.4	18.6	15.4	24.0	28.4	21.7	22.4
